# Short isoform thymic stromal lymphopoietin reduces inflammation and aerobic glycolysis of asthmatic airway epithelium by antagonizing long isoform thymic stromal lymphopoietin

**DOI:** 10.1186/s12931-022-01979-x

**Published:** 2022-03-29

**Authors:** Changhui Yu, Wufeng Huang, Zicong Zhou, Shixiu Liang, Zili Zhou, Jieyi Liu, Haijing Zhao, Laiyu Liu, Hangming Dong, Fei Zou, Shaoxi Cai

**Affiliations:** 1 Department of Respiratory and Critical Care Medicine, Chronic Airways Diseases Laboratory, Nanfang Hospital, Southern Medical University, Guangzhou, 510515 China; 2 Department of Occupational Health and Occupational Medicine, Guangdong Provincial Key Laboratory of Tropical Disease Research, School of Public Health, Southern Medical University, Guangzhou, 510515 Guangdong China

**Keywords:** Asthma, Airway epithelial cells inflammation, Thymic stromal lymphopoietin, Aerobic glycolysis, TSLPR and IL-7R receptor complex

## Abstract

**Background:**

Up-regulation of aerobic glycolysis has been reported as a characterization of asthma and facilitates airway inflammation. We has been previously reported that short isoform thymic stromal lymphopoietin (sTSLP) could reduce inflammation in asthmatic airway epithelial cells. Here we wanted to investigate whether the inhibition of sTSLP on asthma is related to aerobic glycolysis.

**Methods:**

Asthmatic model was established in challenging Male BALB/c mice and 16-HBE (human bronchial epithelial) cell line with house dust mite (HDM). Indicators of glycolysis were assessed to measure whether involve in sTSLP regulating airway epithelial cells inflammation in asthmatic model in vivo and in vitro.

**Results:**

sTSLP decreased inflammation of asthmatic airway and aerobic glycolysis in mice. HDM or long isoform thymic stromal lymphopoietin (lTSLP) promoted HIF-1α expression and aerobic glycolysis by miR-223 to target and inhibit VHL (von Hippel-Lindau) expression 16-HBE. Inhibition of aerobic glycolysis restrained HDM- and lTSLP-induced inflammatory cytokines production. sTSLP along had almost no potential to alter aerobic glycolysis of 16-HBE. But sTSLP decreased LDHA (lactate dehydrogenase A) and LD (Lactic acid) levels in BALF, and HIF-1α and LDHA protein levels in airway epithelial cells of asthma mice model. lTSLP and sTSLP both induced formation of TSLPR and IL-7R receptor complex, and lTSLP obviously facilitated phosphorylation of JAK1, JAK2 and STAT5, while sTSLP induced a little phosphorylation of JAK1 and STAT5.

**Conclusion:**

We identified a novel mechanism that lTSLP could promote inflammatory cytokines production by miR-223/VHL/HIF-1α pathway to upregulate aerobic glycolysis in airway epithelial cells in asthma. This pathway is suppressed by sTSLP through occupying binding site of lTSLP in TSLPR and IL-7R receptor complex.

## Introduction

Airway epithelial cells has been typically considered as the first defensive barrier by preventing allergen invasion [1]. But Airway epithelial cells lost the function to been a generation source of pro-inflammatory cytokine in asthma patient [2]. IL-25, IL-33 and TSLP are the type alarmins cytokine and released mainly by asthma airway epithelial cells [3]. Among them, thymic stromal lymphopoietin (TSLP) is a novel interleukin (IL)-7-like cytokine and could initiate type 2 inflammatory response by TSLPR heterocomplex, which is composed of the IL-7Rα chain and TSLPR chain, in asthma airway [4, 5]. Previous clinical researches reported that blocking TSLP with antibody mitigated airway responsive to allergens, reduced the decline of FEV1 and improved airway inflammation [6, 7].

Recent studies have addressed the two distinct isoforms of TSLP, lTSLP and sTSLP [8, 9]. Our study has observed that lTSLP was significantly increased in asthma airway epithelial cells and promoted pro-inflammatory cytokine production from airway epithelial cells, whereas sTSLP exhibited an inhibitory effect on inflammation and no change in asthma airway epithelial cells expression [10], which is consistent with Maria Rescigno’s report in skin and intestinal epithelial cells [8]. However, the mechanism of sTSLP inhibited-inflammation of asthma airway epithelial cells remains unclear.

Warburg effect (aerobic glycolysis) is the metabolic shift from oxidative phosphorylation to glycolysis in aerobic environment, which is well-known in cancer [11]. Recent studies have found that aerobic glycolysis could also be found in rapidly growing normal cells, lymphocytes and macrophages in immune diseases [12, 13]. Aerobic glycolysis not only provides energy to cells, but also affects cell proliferation, ECM synthesis, autophagy and apoptosis [12, 14–16]. Furthermore, aerobic glycolysis is increased and inhibition of aerobic glycolysis improved airway inflammation and hyperreactivity in a mouse model of asthma [17]. However, there is still a lack of evidence regarding whether aerobic glycolysis is present in airway epithelial cells.

Therefore, in the current study, we intended to examine whether sTSLP regulated aerobic glycolysis participates in regulating inflammatory cytokine production in asthma airway epithelial cells.

## Methods

### Reagents

House dust mites (HDM, ALK-Abello A/S, Denmar), Recombinant Human long-isoform TSLP (lTSLP) was obtained from R&D systems. Synthetic sfTSLP peptides (63aa: MFAMKTKAALAI WCPGYSETQINATQAMKKRRKRKVTTNKCLEQVSQLQGLWRRFNRPLLKQQ) were prepared by China Peptides (Shanghai, China). 3-PO (3-(3-pyridinyl)-1-(4-pyridinyl)-2-propen-1-one) and BAY 87-2243 (Selleck, China). Rabbit anti-HIF-1α, anti-LDHA, anti-PHD and anti-VHL (proteintech, China), Rabbit anti-STAT5, anti-p-STAT5, anti-JAK1, anti-p-JAK1, anti-JAK2and anti-p-JAK2 (Cell Signaling Technology, USA), Rabbit anti-TSLPR and mouse anti-IL-7R (santa curz, USA). Rabbit anti-TSLP (Abcam, USA).

### Animals and experimental protocol

The animal specimens used in this experiment were established as follow, briefly: 30 Male BALB/c mice (6–8 weeks) were purchased from Southern Medical University. The mice were housed in a SPF facility with 12-h dark/light cycles and fed with sterile water and irradiated food. In the first protocol, mice were randomized to the following groups (10 mice/group): in control group: male BALB/c mice were exposed to PBS (100 μL/day/mice). In asthma group: male BALB/c mice were sensitized with intraperitoneal 4000 U HDM (100 μL) on days 1 and 7, then challenged 5 times a week by intranasal (i.n.) instillations of 100 μL HDM for a total of 8 weeks. In Asthma + sTSLP groups, mice were pretreated with sTSLP (1 μg/day/mice) 60 min prior to the administration of HDM. These treatment procedures were carried out daily for 5 consecutive days, followed by two days of rest, for 8 consecutive weeks. Assessment of airway hyper-responsiveness (AHR), pulmonary histologic examination and obtaining of bronchoalveolar lavage fluid (BALF) as described previously [10]. The asthmatic models experiment were approved by the Animal Subjects Committee of South Medical University.

### Cell culture and treatment

RPMI 1640 medium (Gibco) with 10% fetal bovine serum (Gibco, US) was used to culture the human bronchial epithelial cell line, 16-HBEo (16-HBE; Shanghai Fuxiang Biological Technology Co. Ltd., ATCC, USA) and that was placed in a humidified incubator at 37 °C with an atmosphere of 5% CO_2_. Cell processing is detailed in the results and legends.

### Analysis of glucose uptake, lactate dehydrogenase activation, and lactate production

Glucose uptake levels were determined by measuring the uptake of 2-NBDG according to the manufacturer’s instructions (keygen biotech, China). Briefly, 1 × 10^4^ cells/well were plated in 96-well with 100 µL glucose-free culture medium. Ten minutes before the end of the treatment, add 2-NBDG to a final concentration of 100–200 µg/mL in glucose-free medium for 10 min. Aspirate the supernatant and ddd 200 µL of glucose-free culture medium to each well. dFinally, use a fluorescence microplate reader to detect the fluorescence value (excitation/emission = 485/535 nm).

Lactate dehydrogenase (LDHA) activities was determined with an LDHA assay kit according to the manufacturer’s instructions (Jiancheng Bioengineering Institute, Nanjing, China). Briefly, 1 × 106 cells were homogenize on ice in 2 volumes of cold Assay Buffer. Centrifuge cells at 4 °C at 10,000×*g* for 15 min and collect the supernatant. 2 µL LDH Substrate Mix and 48 µL sample were mix, and then incubated reaction for 1 h at 37 °C protected from light. Measure output at OD 450 nm on a microplate reader.

Lactate production levels were determined with an LDH assay kit according to the manufacturer’s instructions (Jiancheng Bioengineering Institute, Nanjing, China). Briefly, 1 × 106 cells were homogenize on ice in 5 volumes of cold Assay Buffer. Centrifuge cells at 4 °C at 10,000×*g* for 5 min and collect the supernatant. 2 µL enzyme mix, 2 µL probe and 46 µL sample were mix, and then incubated reaction for 30 min at room temperature protected from light. Measure output at OD 570 nm on a microplate reader.

### ELISA

ELISA kits (eBioscience, USA) were used for measure serum IgE, IL-4, IL-5 and IL-33 in BALF, IL-25 and IL-33 in cell culture supernatants according to the manufacturer’s instructions.

### mRNA interference

siRNAs targeting TSLP mRNA for downregulating lTSLP protein expression were purchased from Genepharma (Shanghai, China), miRNA mimics targeting to VHL were synthesized in RiboBio (Guangzhou, China) and were transfected into 16-HBE cells using lip-3000 (life, USA) according to the manufacturer’s instructions.

### Western blot analysis

Western blot studies were performed as described previously [10]. The primary antibodies used were shown in reagents. IRDye680 (LI-COR Biosciences, Lincoln, NE) were used as secondary antibodies. Signal intensities were analyzed using an Odyssey Infrared Image System (LI-COR Biosciences).

### Quantitative real-time PCR (RT-PCR)

The extraction of total RNA, reverse transcription of first-strand cDNA and amplification for detecting for mRNA of lTSLP and sTSLP were performed as previously described [10]. The miRNA levels were quantified by qRT-PCR using TaqMan assay kits (ABI) with U6 snRNA as the reference.

### Luciferase assays

The 3′-UTRs of VHL and the mutated 3′-UTRs VHL (MUT) were amplified and inserted downstream from the stop codon of Renilla luciferase using the psiCHECK-2 vector (Sagene, China). 16-HBE cells were cultured in 96-well plates and co-transfected with 10 ng psiCHECK-2-VHL 3′-UTR or psiCHECK-2-MUT 3′-UTR plasmid and 5 pmol mimics-223 or NC. After 48 h of incubation, firefly and Renilla luciferase activities were measured using a Dual-Luciferase Reporter Assay System (Promega, Madison, WI) as described previously [18].

### Immunohistochemistry

Immunohistochemistry (IHC) assays were performed as described previously [19]. In brief, the paraffin sections of lung tissues of asthma mice model were collected for routine IHC staining for LDHA, HIF-1α and VHL.

### Co-immunoprecipitation (Co-IP) assays

Co-IP assays were performed according to the instructions of Protein A/G Magnetic Beads for IP (Biotool, USA) as described previously [20]. In brief, cell lysates were prepared using cell lysis buffer (Beyotime, China) for Western and IP and incubated with the Protein A/G Magnetic Beads-Ab complex that were prepared in advance (5 μg Stim1 antibody plus 50 μL Protein A/G Magnetic Beads) for 4 h at 4 °C. The IP matrix–antibody complex was then washed with elution buffer (0.1–0.2 M Glycine, 0.1–0.5% detergent, pH 2.5–3.1), and protein complexes were eluted and subjected to Western-blot assays.

### Immunofluorescence staining (IF)

Cells were cultured on confocal dish fixed with 4% formaldehyde, permeabilized with 0.1% Triton X-100, and blocked with 5% BSA for 30 min at room temperature. Cells were stained with TSLPR and IL-7R, at a dilution of 1:50 in 5% BSA overnight at 4 °C, before incubated with Alexa Fluor 488 (R37118) or Alexa Fluor 594 (R37119) (1:200 diluted in PBS) (Invitrogen, USA) at room temperature for 1 h. After that, cells were incubated with DAPI (1:1000 diluted in PBS) for 5 min before laser scanning under a confocal microscope (FV1000, Olympus) at 100× objective magnification.

### Statistical analysis

Statistical analysis was carried out using the SPSS (version 19.0) software package. The variables were expressed as the mean ± standard deviation (SD). One-way ANOVA accompanied by Bonferonni’s post hoc test for multiple comparisons were utilized to compare differences between groups. Values of *P* < 0.05 were considered to be statistically significant.

## Results

### sTSLP decreased inflammation of airway and aerobic glycolysis in asthmatic mice

**Fig 1:**
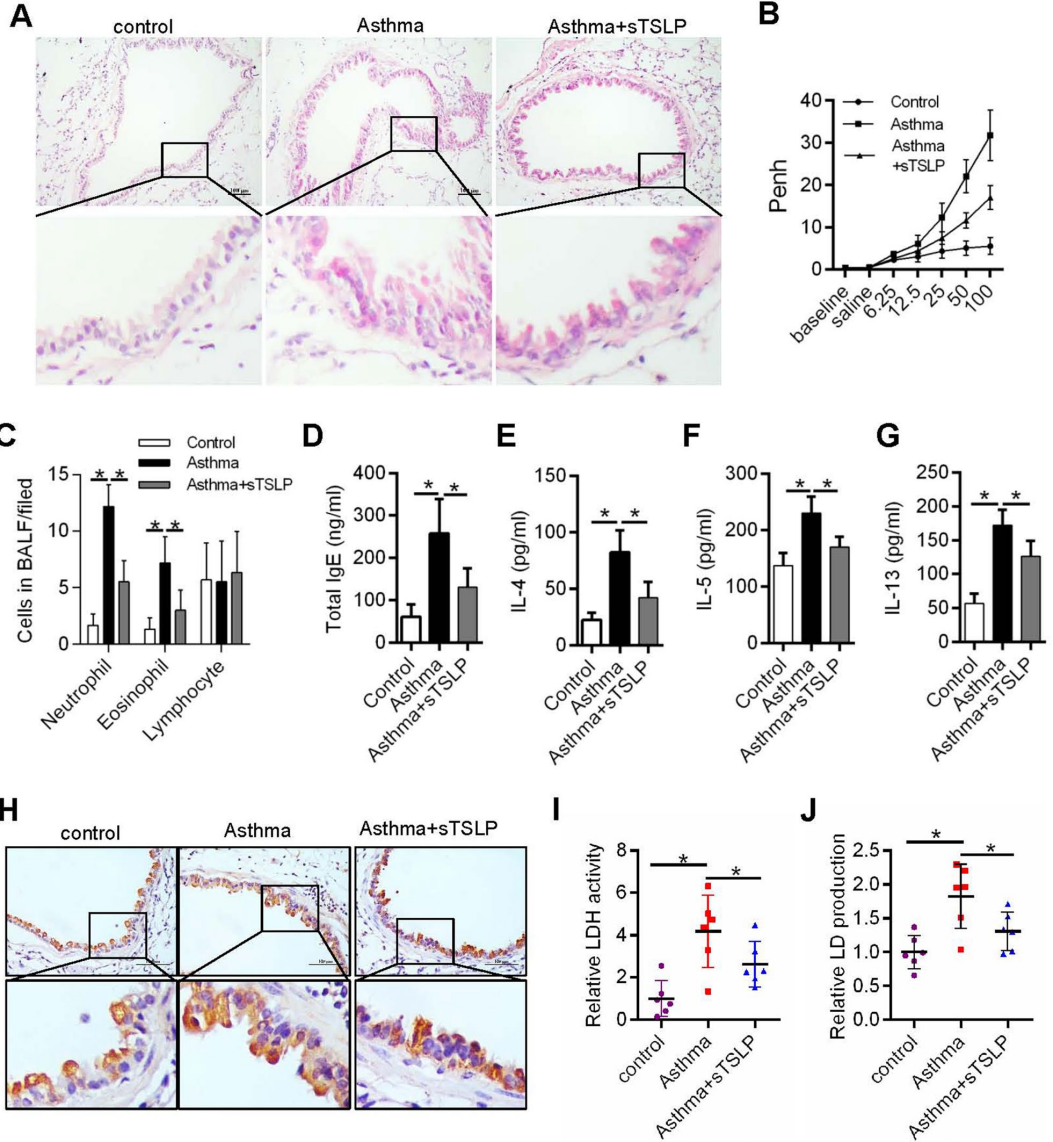
sTSLP ameliorated inflammation of airway and aerobic glycolysis in asthmatic mice. **A** Representative hematoxylin/eosin-stained lung sections from asthmatic mice models. **B** Airway hyper-responsiveness was measure after methacholine challenge. **C** Differential cell percentages in BALF. ELISA was used to measure the levels of **D** total IgE, **E** IL-4, **F** IL-5 and **G** IL-13 in BALF. **H** Immunohistochemical stains of representative samples of asthma mouse models lung sections showing LDHA expression, scale bars (top row): 100 μm. **I** LDHA activity and **J** LD production levels in BALF of asthma mouse models were measure. *P < 0.05, n = 3 independent experiments

To assess the effect of sTSLP in asthma, pulmonary pathology, AHR and BALF of athematic mice were detected. Treatment with sTSLP significantly decreased inflammation and AHR of airway and neutrophils and eosinophilsin of BALF in mice that inhaled methacholine (Fig. 1A–C). Next, Elevated serum total IgE levels, as marker for asthma, was obviously decreased in sTSLP group, as well as levels of Th2-associated cytokines (IL-4, IL-5 and IL-13) in the BALF of mice (Fig. 1D–G). Aerobic glycolysis has been reported to be involved in asthma program by activating T cell [17]. To test whether aerobic glycolysis plays a role in sTSLP ameliorating asthmatic airway inflammation. The LDHA, a key for aerobic glycolysis in cell cytoplasm, expression in airway epithelium of asthmatic mice, and LDH activity and LD production levels in BALF were significantly decreased in asthmatic mice with sTSLP treatment (Fig. 1H–J). These data implied that sTSLP may be reduced inflammation through inhibited aerobic glycolysis in asthmatic airway epithelium.

### Aerobic glycolysis was involved in HDM/lTSLP-induced inflammatory cytokine release of airway epithelium

**Fig. 2:**
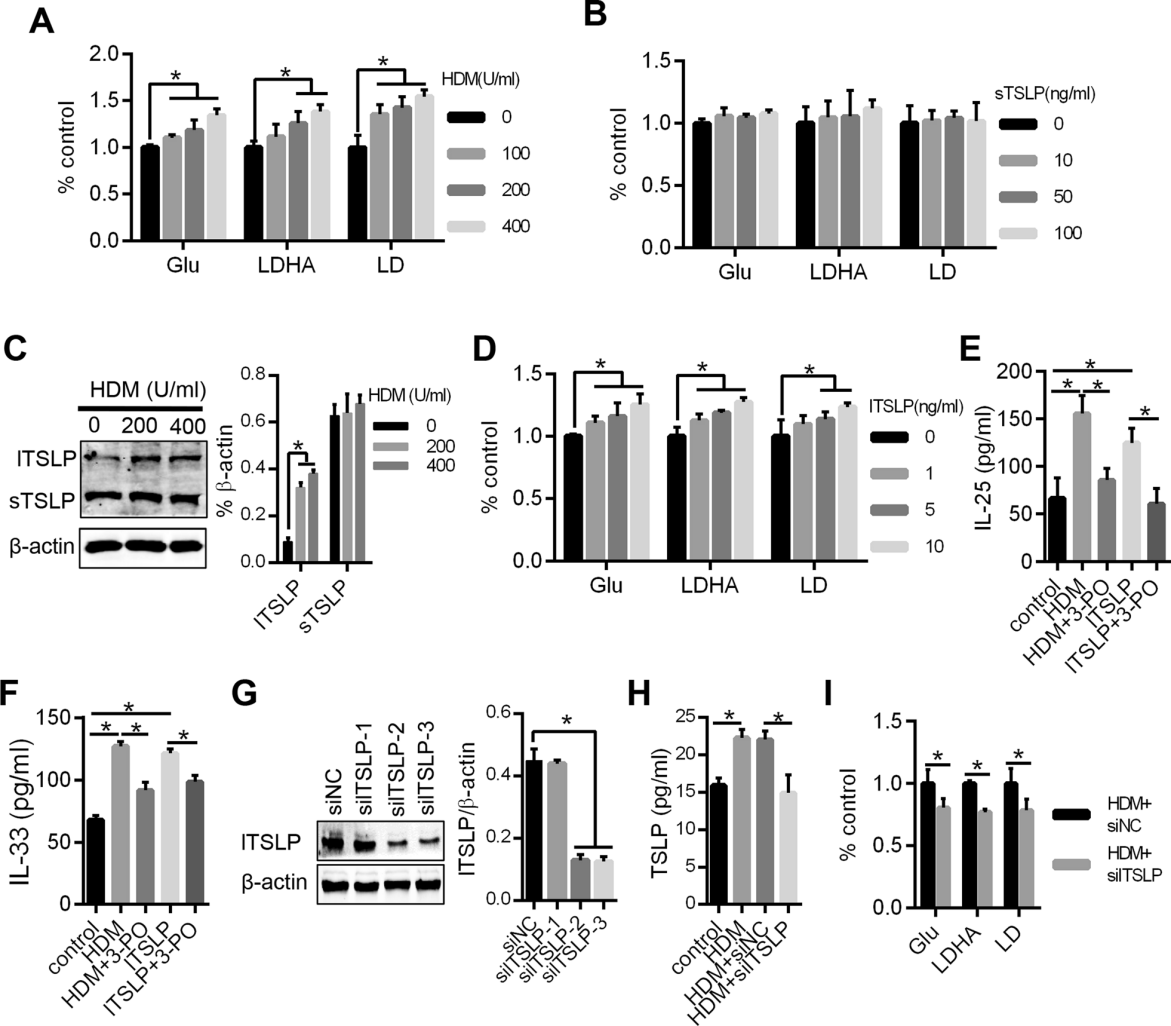
Aerobic glycolysis was involved in HDM/lTSLP-induced TH2 Inflammatory cytokine release of airway epithelium. Airway epithelium 16-HBE was treatment with different concentration of HDM (**A**) and sTSLP (**B**) for 24 h and the indicators of aerobic glycolysis: glucose uptake (Glu), lactate dehydrogenase activity (LDHA) and lactate production (LD) were detected. **C** The expression levels of lTSLP and sTSLP protein were detected by western blot in HDM-induced 16-HBE. **D** The Glu, LDHA and LD were measured in 16-HBE with lTSLP treatment. **E**, **F** ELISA was used to measure inflammatory cytokine IL-25/33 in cell culture supernatant. **G** Western blots analysis showing lTSLP levels after lTSLP siRNA transfection in 16-HBE. **H** lTSLP levels in cell culture supernatant were measured by ELISA. **I** A series of metabolic parameters was measured for Glu, LDHA and LD in 16-HBE with lTSLP knockdown. *P < 0.05, n = 3 independent experiments

To clear whether sTSLP decreased inflammation through inhibited aerobic glycolysis, we firstly explored the effect of aerobic glycolysis role in inflammation of airway epithelium in asthma. We measured the glycolysis of 16-HBE grown in HDM or sTSLP under normal oxygen conditions, and found that HDM induced a growing glucose uptake, lactate dehydrogenase activity and lactate production, which representing glycolysis levels, but no obvious change in sTSLP group was observed (Fig. 2A, B). Meantime, we assessed the expression difference of lTSLP and sTSLP in asthma airway epithelial cells model, HDM justly promoted lTSLP protein expression (Fig. 2C). lTSLP could enhanced glycolysis of 16-HBE (Fig. 2D). In addition, inhibition of glycolysis significantly decreased inflammatory cytokines levels (IL-25 and IL-33) in 16-HBE with HDM and lTSLP treatment (Fig. 2E, F). Moreover, after knockdown expression of lTSLP, HDM-induced increased glycolysis was reduced (Fig. 2G, I). These results indicated that HDM/lTSLP could promote inflammatory cytokines production by enhancing aerobic glycolysis in asthma airway epithelium.

### HDM/lTSLP activated HIF-1α/LDHA pathway by restrain VHL expression

**Fig. 3:**
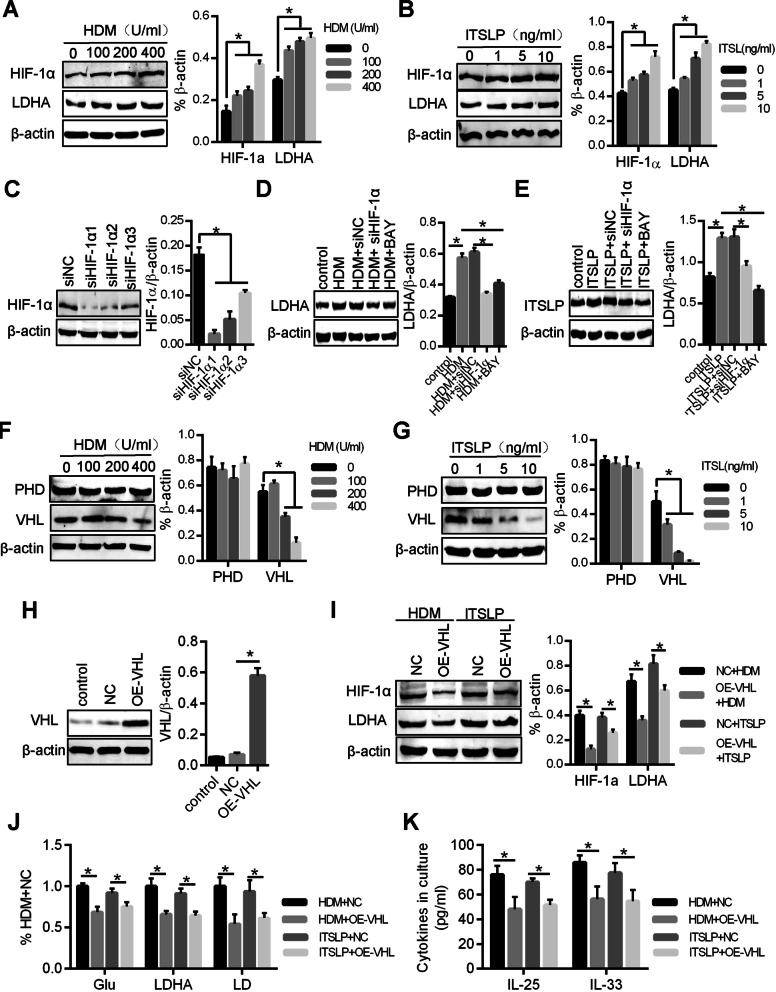
HDM/lTSLP activated HIF-1α/LDHA pathway by restrain VHL expression. Western blots analysis showing HIF-1α and LDHA levels in 16-HBE was treated with different concentration of HDM (**A**) and lTSLP (**B**). **C** HIF-1α expression was knockdown by siRNA and was measure by western blots. After knockdown of HIF-1α expression and pretreatment with HIF-1α inhibitor BAY-872247 (BAY), HDM- (**D**) or lTSLP- (**E**) induced LDHA expression were measured by western blots. Western blots analysis showing PHD and VHL levels in 16-HBE was treated with different concentration of HDM (**F**) and lTSLP (**G**). **H** 16-HBE was infected with lentivirus to overexpress VHL (OE-VHL). **I** After overexpression of VHL, HDM (400 U/ml)- or lTSLP (10 ng/ml)-induced HIF-1α and LDHA expression were measure by western blot assay. **J** A series of metabolic parameters was measured for Glu, LDHA and LD in 16-HBE with VHL overexpression. **K** ELISA was used to measure inflammatory cytokine IL-25 and IL-33 in cell culture supernatant. *P < 0.05, n = 3 independent experiments

Hypoxia inducible cytokine-1α (HIF-1α) is a key regulatory cytokine in aerobic glycolysis by facilitating expression of glucose transporter proteins, hexokinase and LDHA etc. [11]. We examined whether highly expressed HIF-1α in 16-HBE would grow with HDM or lTSLP. To this goal, 16-HBE was stimulated with HDM or lTSLP under different concentrations. The results indicated that expression of HIF-1α and its downstream protein LDHA, an import cytokine for glycolysis, showed concentration-dependent increase (Fig. 3A, B). Furthermore, inhibition or knockdown expression of HIF-1α could restrain HDM and lTSLP to promote LDHA expression (Fig. 3C–E).

HIF-1α would be hydrolyzed in aerobic environment by proline hydroxylase (PHD) and VHL [21, 22], but it is highly expressed in HDM and lTSLP-stimulated airway epithelium under normoxic conditions. Interestingly, HDM and lTSLP could suppress VHL expression, but not altering the expression of PHD (Fig. 3F, G). Contrarily, HDM and lTSLP lost the potential to promote HIF-1α and LDHA expression, glycolysis and inflammatory cytokines (IL-25 and IL-33) production in 16-HBE with VHL overexpression (Fig. 3H–K). Thus, HIF-1α is the critical protein in HDM and lTSLP-induced aerobic glycolysis and inflammatory cytokines levels of airway epithelium.

### miR-223 mediated HDM/lTSLP-induced HIF-1α expression by targeting VHL

**Fig. 4:**
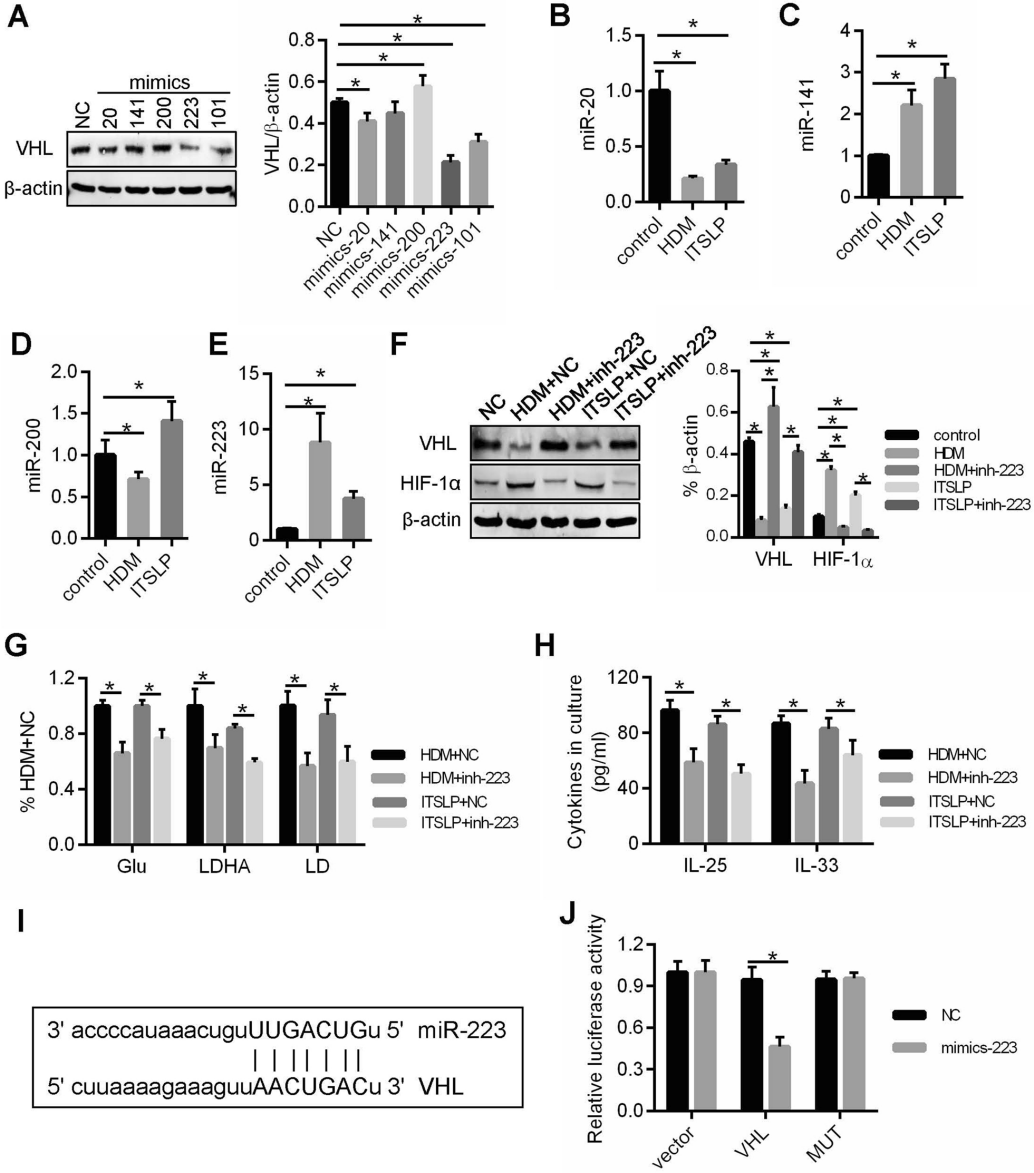
miR-223 mediated lTSLP-induced HIF-1α expression by targeting VHL. **A** Western blots showing VHL protein expression in 16-HBE after miRNAs overexpression by infecting with five different miRNAs mimics. **B**–**E** the expression of miR-20, miR-141, miR-200 and miR-223 in HDM- or lTSLP-induced 16-HBE was detected by quantitative polymerase chain reaction analysis. **F** Western blots showing VHL and HIF-1α expression in 16-HBE expressing miR-223 inhibitor (inh-223) and treating with HDM or lTSLP. **G** The Glu, LDHA and LD were measured in 16-HBE with inh-223 transfection. **H** ELISA was used to measure inflammatory cytokine IL-25 and IL-33 in cell culture supernatant. **I** Sequences present in the 3′-UTR of VHL targeted by miR-223 and its target region are capital. **J** The effect of miR-223 expression on luciferase reporter gene activity when linked with the targeted segment of the 3′-UTR of VHL by luciferase assay. *P < 0.05, n = 3 independent experiments

We next explored how HDM- or lTSLP-induced VHL low expression in 16-HBE. We screened the output of prediction algorithms (http://www.microrna.org/) to determine whether miRNAs could regulate VHL expression and according to Prescott’s [23] report about the miRNAs change in airway epithelium of asthma patient, four miRNAs (miR-20, miR-141, miR-200 and miR-223) may target VHL. Firstly, fives mimics of miR-20, miR-141, miR-200, miR-223 and miR-101 (miR-101 has been confirmed to target VHL [24]) that were transfected into 16-HBE, and then we found that miR-20 and miR-223 could significantly reduce VHL expression as miR-101 (Fig. 4A). However, miR-223 was both notably increased in HDM- or lTSLP-stimulated airway epithelium, but not miR-20 (Fig. 4B–E). Moreover, inhibitor of miR-223 was transfected to 16-HBE respectively, resulting in HDM- or lTSLP-induced VHL low expression, HIF-1α expression, glycolysis and inflammatory cytokines (IL-25 and IL-33) production increased were reversed (Fig. 4F–H). We therefore selected miR-223 for further study.

To obtain additional direct evidence that HDM or lTSLP inhibited VHL expression by miR-223, we identified the binding sites for miR-223 in the 3′-UTR of VHL mRNA (Fig. 4I) and created luciferase reporters to measure the direct interactions of miR-223 and VHL mRNA. VHL 3′-UTR reporter or mutant reporters (MUT) and mimics-223 or NC were co-transfected into 16-HBE. The luciferase activity of VHL was lower in the presence of mimics-223 than in that of NC (Fig. 4J). But no notables change was found in luciferase activity for MUT in the presence of mimics-223 or NC (Fig. 4H). Our data illustrated that VHL mRNA is a direct physical target of miR-223.

### sTSLP restrain aerobic glycolysis induced by HDM or lTSLP

**Fig. 5:**
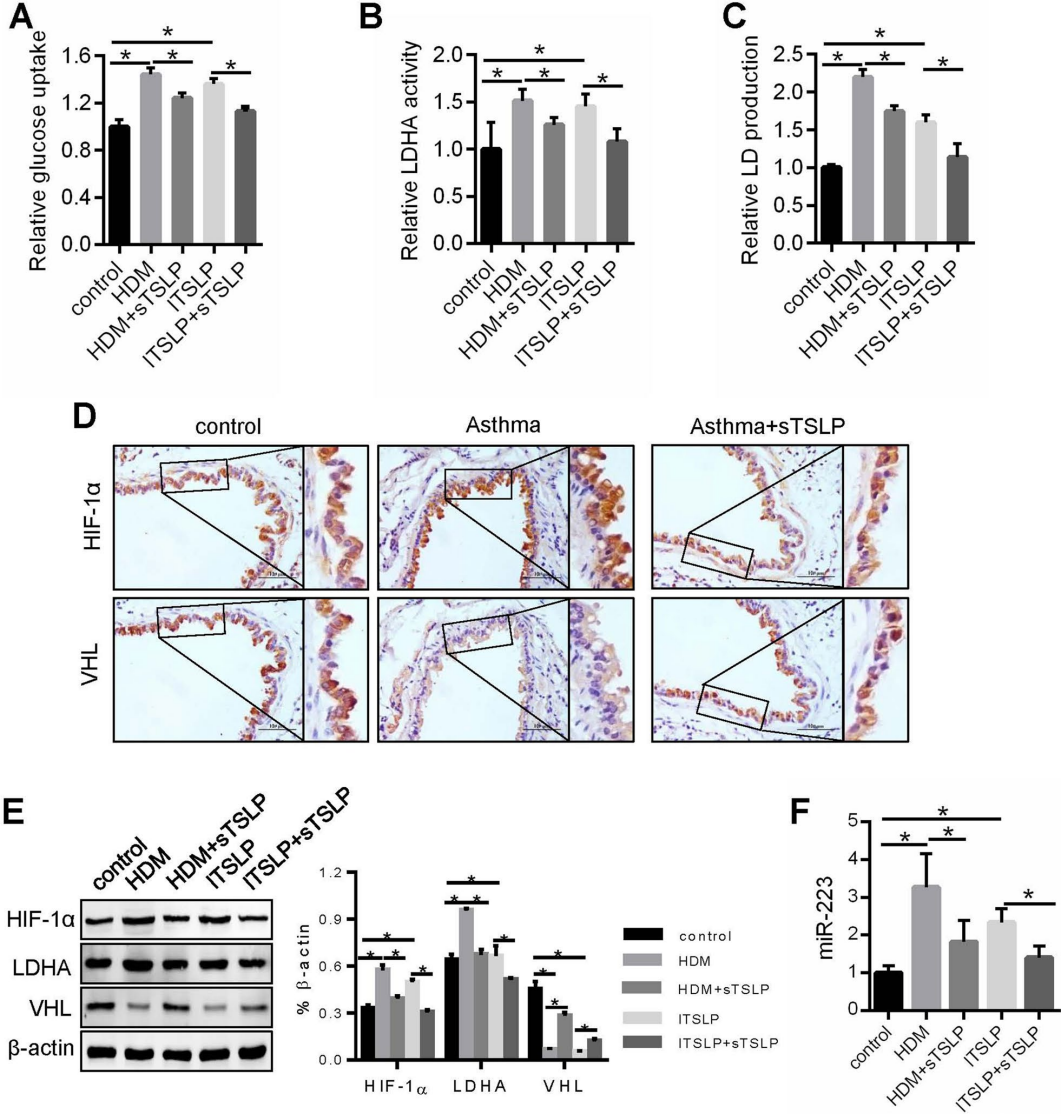
sTSLP restrain aerobic glycolysis induced by HDM or lTSLP in vivo and in vitro. **A**–**C** A series of metabolic parameters was measured for Glu, LDHA and LD in16-HBE. **D** HIF-1α and VHL expression were measure by Immunohistochemical stains of continuous sectioning of lung tissue. **E** Western blot assay showing HIF-1α, LDHA and VHL expression in 16-HBE. **F** Q-PCR was used to analysis the expression of miR-223. *P < 0.05, n = 3 independent experiments

We have observed that sTSLP suppressed glycolysis of asthmatic mice (Fig. 1H, J) and our group has found that sTSLP could improve HDM-induced asthmatic airway epithelial cells inflammation by hampering the effects of lTSLP [10]. So we evaluated that the effects of sTSLP in HDM- or lTSLP-induced glycolysis in 16-HBE, and found HDM- or lTSLP-induced aerobic glycolysis were inhibited by sTSLP (Fig. 5A–C). We next determined the effects of sTSLP on HIF-1α, LDHA, miR-223 and VHL expression. The results showed that sTSLP decreased the HIF-1α, LDHA and miR-223 expression, while prevent VHL in airway epithelium of asthmatic mice, or HDM- or lTSLP-stimulated 16-HBE (Fig. 5D–F). These data suggested that sTSLP could restrain aerobic glycolysis induced by HDM or lTSLP.

### sTSLP reduced HDM-induced aerobic glycolysis by decreasing lTSLP activated-phosphorylation of STAT5

**Fig. 6:**
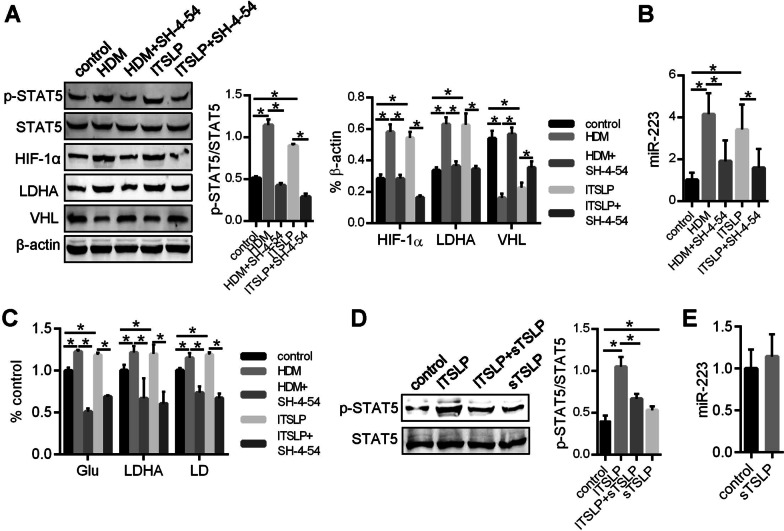
sTSLP reduced lTSLP-induced aerobic glycolysis by inhibiting phosphorylation of STAT5. **A** Western blot assay showing phosphorylation of STAT5 and expression of HIF-1α, LDHA and VHL in 16-HBE. **B** Q-PCR was used to analysis the expression of miR-223. **C** A series of metabolic parameters was measured for glucose uptake (Glu), lactate dehydrogenase activity (LDHA) and lactate production (LD). **D** Western blots showing phosphorylation of STAT5. **E** Q-PCR was used to analysis the expression of miR-223. *P < 0.05, n = 3 independent experiments

It is known that the STAT5 is the main signal pathway of lTSLP, but the importance of this signaling pathway on lTSLP-induced aerobic glycolysis remains unclear. SH-4-54, an inhibitor of STAT5, was used to pre-stimulated 16-HBE before adding HDM or lTSLP. These two could lead to a significantly decreased in the expression of HIF-1α, LDHA, miR-223 and aerobic glycolysis, except VHL (Fig. 6A–C). These data indicated that HDM/lTSLP activated STAT5 to promote miR-223, HIF-1α and LDHA expression. Furtherly, we observed that sTSLP suppressed lTSLP-elevated phosphorylation of STAT5. However, sTSLP also induced a low phosphorylation of STAT5 (Fig. 6D) that was not sufficient to significantly increase miR-223 expression (Fig. 6E). Why sTSLP has dual effects on phosphorylation of STAT5.

### sTSLP induced receptor complex formation of IL-7R and TSLPR as an antagonist to blocking signal from the lTSLP

**Fig. 7:**
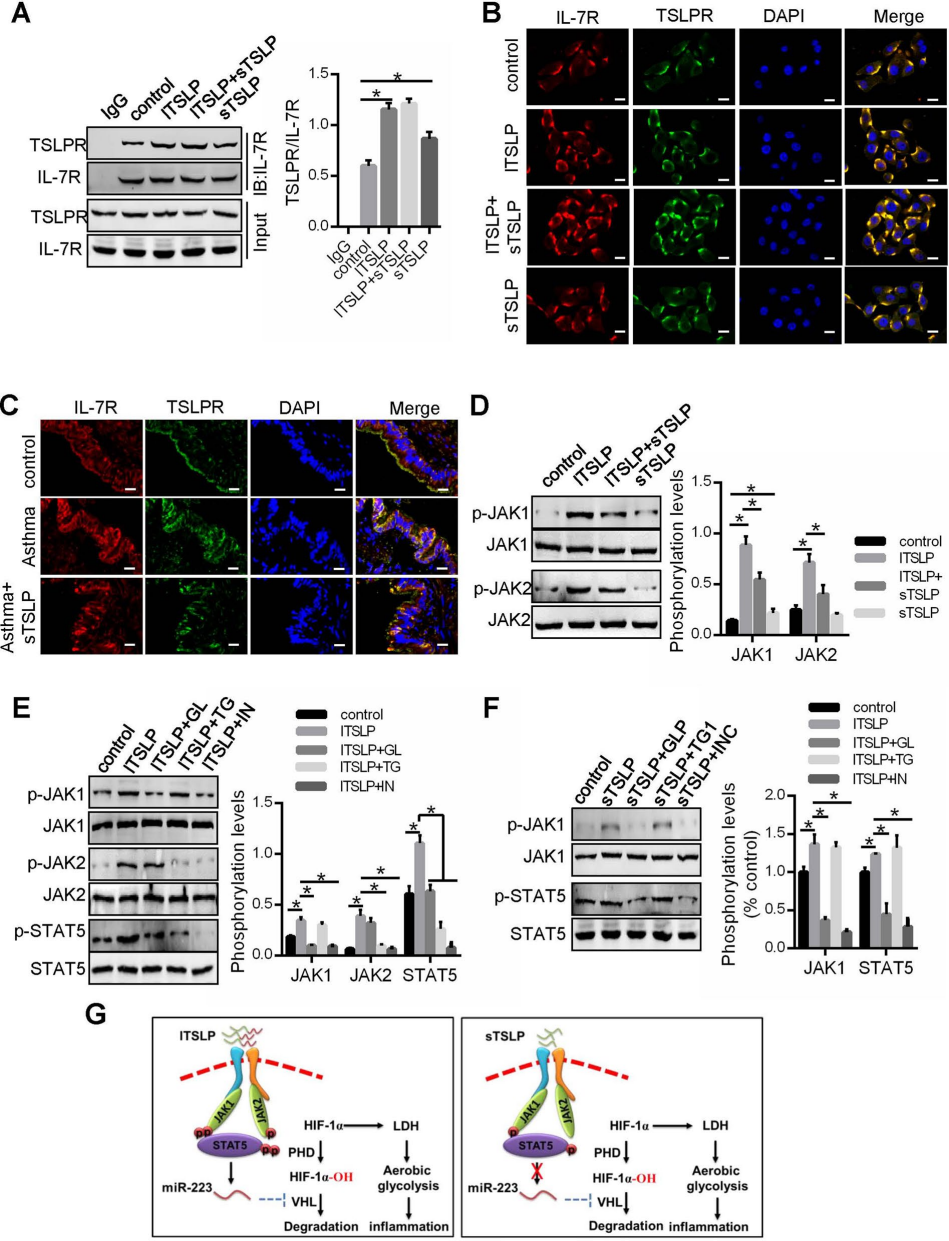
sTSLP induced receptor complex formation of IL-7R and TSLPR as an antagonist to block signal from the lTSLP. **A** Lysates from16-HBE was immunoprecipitated with antibodies to IL-7R, and immunocomplexes were measure by western blot assay. Dual IF staining of IL-7R (red) and TSLPR (green) were performed to analyze interaction of IL-7R and TSLPR in 16-HBE (**B**) and epithelium of asthma mouse airway (**C**). Scale bars: 10 μm. **D** The phosphorylation of JAK1 and JAK2 determined by Western blot. **E**, **F** GLPG0634 (GL, a selective inhibitor of JAK1), TG101348 (TG, a selective inhibitor of JAK2) and INCB018424 (IN, a selective inhibitor of JAK1 and 2) was added respectively into 16-HBE before lTSLP or sTSLP, and phosphorylation of JAK1, JAK2 or STAT5 were determined by Western blot. **G** Schematic model for the different effects of lTSLP and sTSLP on IL-7R/TSLPR/JAK/STAT5 pathway. *P < 0.05, n = 3 independent experiments

Maria Rescigno and others suspected the possibility of sTSLP binding to TSLPR and acting as an antagonist to block signal from the lTSLP [8]. Our results also observed that sTSLP recruited the receptor complexes form of TSLPR and IL-7R, like as lTSLP (Fig. 7A, B). When sTSLP was added into 16-HBE before, the receptor complexes form of TSLPR and IL-7R still increased (Fig. 7A, B). Furthermore, increased receptor complexes form of TSLPR and IL-7R in airway epithelial cells asthma group hasn’t been blocked in sTSLP treatment group (Fig. 7C). These results hinted that sTSLP and lTSLP both induced the receptor complexes form of TSLPR and IL-7R.

But, we assessed the JAK1 and JAK2 activation which is required for TSLPR and IL-7R stimulating STAT5 [25], and found that lTSLP could induced phosphorylation of JAK1 and JAK2, while sTSLP only induced a low level phosphorylation of JAK1 and also decreased lTSLP-induced phosphorylation of JAK1 and JAK2 (Fig. 7D). After phosphorylation of JAK1 and JAK2 inhibition, lTSLP-induced phosphorylation of STAT5 was abolished (Fig. 7E). However, sTSLP-stimulated low level phosphorylation of STAT5 was revered only by JAK1 inhibitor (Fig. 7F). So, these results indicated that despite sTLPS could promotes the receptor complexes form of TSLPR and IL-7R, only slightly activated JAK1-STAT5 pathway, and sTLPS may played an antagonism through occupying the site of lTSLP in the receptor complexes of TSLPR and IL-7R to mitigate JAK1/JAK2-STAT5 pathway.

## Discussion

In the present study, we found that lTSLP promoted pro-inflammatory cytokines production of airway epithelial cells by increasing aerobic glycolysis. Furthermore, lTSLP activated JAK1/JAK2/STAT5 signaling pathway through TSLPR/IL-7R complex, and then promoted miR-223 expression to target inhibition of VHL expression, thereby maintaining HIF-1α Stable to increase aerobic glycolysis in airway epithelium. Simultaneously, we also found that sTSLP had little effect on aerobic glycolysis when stimulating airway epithelium alone, but could inhibit lTSLP-induced aerobic glycolysis by occupying the site of lTSLP in TSLPR/IL-7R complex without activating STAT5 signaling pathway.

Glycolysis generally occurs in an oxygen-deficient environment, providing the necessary ATP to tissues or cells. However, in an aerobic environment, a large amount of glycolysis occurs in tissues or cells that proliferate or metabolize faster, especially tumor cells, which is aerobic glycolysis or Warburg effects [26]. An important reason is that glycolysis produces DNA and other raw materials, and it also provides acidic microenvironment to meet proliferation, invasion or inflammatory cytokine production [12, 27, 28]. Our study found a significant increase in aerobic glycolysis in both the HDM-induced asthma airway epithelial cells model and the asthma mouse model, which is consistent with the Rafeul Alam’s research [17]. Our further studies revealed that the lTSLP-stimulated airway epithelium had increased aerobic glycolysis in the normoxic environment, but the effect of sTSLP is not obvious. Furthermore, inhibition of aerobic glycolysis could reduce the pro-inflammatory cytokines production induced by HDM or lTSLP. It was suggested that lTSLP can induce the pro-inflammatory cytokines production by promoting aerobic glycolysis of airway epithelium.

In glycolysis, HIF-1α is an important regulatory protein that promotes the expression of key enzymes such as GULT1, HK and LDHA, and also promotes pyruvate dehydrogenase kinase (PDK) expression to suppress PDH which is required for tricarboxylic acid cycle [11, 29]. In airway epithelium cultured with lTSLP, we observed significant HIF-1α expression elevation, and HIF-1α inhibition reduced the lTSLP effects. However, in an aerobic environment, HIF-1α is proteolytically hydrolyzed by VHL after being hydroxylated by PHD [30–32]. Interestingly, we found that lTSLP inhibited VHL expression, and overexpression of VHL reduced lTSLP-induced HIF-1α expression. It is suggested that lTSLP may stabilize the expression of HIF-1α to, promoting aerobic glycolysis by inhibiting VHL expression.

How is VHL expression suppressed by lTSLP? miRNAs are important mechanisms regulating protein expression. Four miRNAs, miR-20, miR-141, miR-200 and miR-223, were screened by the miRNA information website (www.microrna.org/) in combination with high-throughput results from Arron [23]. Through a series of studies, we found that lTSLP promoted airway epithelial cells miR-223 expression to target inhibition of VHL expression.

The amino acid series of sTSLP is 40% identical to the C-terminus of lTSLP. We have found that sTSLP attenuates chronic airway inflammation and airway epithelial cells inflammatory cytokine production in asthmatic mice by inhibiting lTSLP induced airway epithelial cells inflammation [10]. In this present study, airway epithelial cells LDHA and HIF-1α expression in asthmatic mice, and lactate dehydrogenase and lactic acid in alveolar lavage fluid were found obviously downregulated in asthma mice with sTSLP treatment. Further in airway epithelium, sTSLP was also observed to reduce HDM and lTSLP-induced aerobic glycolysis, and expression of LDHA and HIF-1α.

In skin and intestinal inflammation studies, Maria Rescigno et al. [8] speculated that although sTSLP hasn’t the lTSLP N-terminal structure and cannot bind to TSLPR, it may act as an antagonist to inhibit lTSLP binding to its receptor complex TSLPR/IL-7. By co-immunoprecipitation and immunofluorescence staining, we did observe that sTSLP induced formation of complex TSLPR/IL-7 and activated JAK1 and STAT5 slightly (no significant), but no effect to JAK2. This illustrates that sTSLP blocks the function of lTSLP by antagonizing lTSLP activating TSLPR/IL-7 and downstream JAK1/JAK2/STAT5.

## Conclusion

In summary, airway epithelial cellsaerobic glycolysis participates in the formation of chronic inflammatory cytokines and promotes chronic inflammatory response in asthmatic airways. lTSLP and sTSLP, a pair of homologous and different cytokines, regulated the ability of HIF-1α to promote aerobic glycolysis through different functions of TSLPR/IL-7 complex in airway epithelium. Through the current study, the role and mechanism of lTSLP and sTSLP in asthma are further clarified, which may provide new ideas for the diagnosis and treatment of asthma.

## Data Availability

All data generated or analysed during this study are included in this article.

## Abbreviations

sTSLP: Short isoform thymic stromal lymphopoietin
HDM: House dust mite
16-HBE: Human bronchial epithelial cells
lTSLP: Long isoform thymic stromal lymphopoietin
VHL: Von Hippel-Lindau
LDHA: Lactate dehydrogenase A
LD: Lactic acid
TSLP: Thymic stromal lymphopoietin
IL: Interleukin
AHR: Airway hyper-responsiveness
BALF: Bronchoalveolar lavage fluid
IHC: Immunohistochemistry
Co-IP: Co-immunoprecipitation
IF: Immunofluorescence staining
PHD: Proline hydroxylase
PDK: Pyruvate dehydrogenase kinase
